# Introduction to the Coproduction of Supervision Standards for Digital Peer Support: Qualitative Study

**DOI:** 10.2196/40607

**Published:** 2023-06-19

**Authors:** Caroline Collins-Pisano, Michael Johnson, George Mois, Jessica Brooks, Amanda Myers, Deanna Mazina, Marianne Storm, Maggie Wright, Nancy Berger, Ann Kasper, Anthony Fox, Sandi MacDonald, Sarah Schultze, Andrew Bohm, Julia Hill, Karen Fortuna

**Affiliations:** 1 Department of Psychiatry Dartmouth College Hanover, NH United States; 2 The Commission on Accreditation of Rehabilitation Facilities International Tucson, AZ United States; 3 College of Applied Health Sciences University of Illinois Champaign, IL United States; 4 Department of Psychiatry School of Medicine and Public Health University of Wisconsin–Madison Madison, WI United States; 5 The Heller School for Social Policy and Management Brandeis University Waltham, MA United States; 6 Watertown Health Department Watertown, MA United States; 7 Faculty of Health Sciences and Social Care Molde University College Molde Norway; 8 Families in Trauma and Recovery Fife United Kingdom; 9 Roxbury Community College Boston, MA United States; 10 Kasper Connects Portland, OR United States; 11 Tennessee Mental Health Consumer's Association Nashville, TN United States; 12 International Association of Pre-Menstrual Disorders Boston, MA United States; 13 School of Social Work University of New Hampshire Durham, NH United States; 14 The Dartmouth Institute for Health Policy and Clinical Practice Geisel School of Medicine Dartmouth College Hanover, NH United States; 15 Dartmouth College Hanover, NH United States; 16 Department of Psychiatry Geisel School of Medicine Dartmouth College Concord, NH United States

**Keywords:** digital peer support, mHealth, standards, guideline, peer support, supervision, focus group, qualitative data analysis, competency, competencies, health care education, professional education, professional development, continuing education

## Abstract

**Background:**

Digital peer support enhances engagement in mental and physical health services despite barriers such as location, transportation, and other accessibility constraints. Digital peer support involves live or automated peer support services delivered through technology media such as peer-to-peer networks, smartphone apps, and asynchronous and synchronous technologies. Supervision standards for digital peer support can determine important administrative, educative, and supportive guidelines for supervisors to maintain the practice of competent digital peer support, develop knowledgeable and skilled digital peer support specialists, clarify the role and responsibility of digital peer support specialists, and support specialists in both an emotional and developmental capacity.

**Objective:**

Although digital peer support has expanded recently, there are no formal digital supervision standards. The aim of this study is to inform the development of supervision standards for digital peer support and introduce guidelines that supervisors can use to support, guide, and develop competencies in digital peer support specialists.

**Methods:**

Peer support specialists that currently offer digital peer support services were recruited via an international email listserv of 1500 peer support specialists. Four 1-hour focus groups, with a total of 59 participants, took place in October 2020. Researchers used Rapid and Rigorous Qualitative Data Analysis methods. Researchers presented data transcripts to focus group participants for feedback and to determine if the researcher’s interpretation of the data match their intended meanings.

**Results:**

We identified 51 codes and 11 themes related to the development of supervision standards for digital peer support. Themes included (1) education on technology competency (43/197, 21.8%), (2) education on privacy, security, and confidentiality in digital devices and platforms (33/197, 16.8%), (3) education on peer support competencies and how they relate to digital peer support (25/197, 12.7%), (4) administrative guidelines (21/197, 10.7%), (5) education on the digital delivery of peer support (18/197, 9.1%), (6) education on technology access (17/197, 8.6%), (7) supervisor support of work-life balance (17/197, 8.6%), (8) emotional support (9/197, 4.6%), (9) administrative documentation (6/197, 3%), (10) education on suicide and crisis intervention (5/197, 2.5%), and (11) feedback (3/197, 1.5%).

**Conclusions:**

Currently, supervision standards from the Substance Abuse and Mental Health Services Administration (SAMHSA) for in-person peer support include administrative, educative, and supportive functions. However, digital peer support has necessitated supervision standard subthemes such as education on technology and privacy, support of work-life balance, and emotional support. Lack of digital supervision standards may lead to a breach in ethics and confidentiality, workforce stress, loss of productivity, loss of boundaries, and ineffectively serving users who participate in digital peer support services. Digital peer support specialists require specific knowledge and skills to communicate with service users and deliver peer support effectively, while supervisors require new knowledge and skills to effectively develop, support, and manage the digital peer support role.

## Introduction

Digital peer support may show longevity past the COVID-19 era. In April 2020, a survey of 180 peer support specialists from 23 states found a 95% increase in peer support specialists offering digital peer support and a 90% increase in peer support specialists’ confidence in digital peer support [1]. Peer support has transformed to be offered through digital technologies and telemental health sessions [1]. Digital peer support involves live or automated peer support services delivered through technology media such as peer-to-peer networks, smartphone apps, and asynchronous and synchronous technologies [2]. Peer support has been described as social-emotional support, frequently coupled with instrumental support [3]. It is often provided by persons with a lived experience of a mental health condition or substance use disorder to others sharing a similar mental health condition and substance use disorder or mutually offered between both people. The World Health Organization defines peer support as an essential mental health service [4].

Digital peer support expands the reach and practices of in-person peer support and enhances service users’ abilities to engage in mental and physical health services despite barriers such as location, transportation, and other accessibility constraints. Digital peer support sessions have no geographic or time limitations, promote high levels of engagement when developed with peer support specialists as partners, engage service users in mental health services outside of clinical settings, and have access to harder-to-reach groups such as rural residents, older adults, and people experiencing homelessness [5]. Like in-person peer support, digital peer support enhances the quality of life, functioning, social support, recovery, hope, and empowerment. Studies on the feasibility and preliminary effectiveness have found that digital peer support services reduce mental health symptoms and promote engagement in services [5].

Although digital peer support has gained traction globally, at present, no formal digital supervision standards have been put in place. Supervision is considered critical for the development of competent mental health workers [6]. Supervision standards have the potential to aid in the transition to telemental health delivery and help telehealth workers to develop the competencies needed for the telemental health services [6]. According to the Substance Abuse and Mental Health Services Administration (SAMHSA), supervision is a collaborative activity between a supervisor and their workers in which the supervisor guides and supports the worker to promote the fidelity-adherent delivery of services and the development of the worker’s skills and knowledge surrounding the peer support [7]. SAMHSA is an agency within the United States Department of Health and Human Services, which leads public health efforts to advance the behavioral health of the United States.

Supervision of peer support specialists can help to enhance problem-solving skills, improve clarity in the decision-making process, empower workers, increase satisfaction, and help peer support specialists to deliver better services due to opportunities for reflection and discussion of work and work-related issues [8]. Effective supervision practices can also help mental health organizations to better manage resources, improve performance, and increase morale [8].

The transition to, and maintenance of, digital peer support offers challenges for both peer support specialists and service users. For example, a recent study about combining web-based and offline peer support discusses challenges related to the transition to digital peer support, such as protecting confidentiality of service users [9]. Similar to other fields, supervision standards can assist in the development of knowledgeable and skilled digital peer support specialists to clarify the role and responsibility of digital peer support specialists and support specialists in both an emotional and developmental capacity. Introducing supervision standards for digital peer support has the potential to help determine important administrative, educative, and supportive guidelines that supervisors could use to maintain the practice of competent digital peer support. As such, the aim of this study is to inform the development of supervision standards for digital peer support and introduce guidelines that supervisors can utilize to support, guide, and develop competencies in digital peer support specialists.

## Methods

### Measures

Peer support specialists that currently offer digital peer support services were recruited via email by an international listserv of 1500 peer support specialists, using a convenience sampling method. Participants were eligible if they were (1) 18 years of age or older and (2) a peer support specialist. Participants were asked to complete a web-based presurvey with questions on demographic information (eg, age, race, and gender) to ensure variation in focus group participants and participated in a 1-hour web-based focus group via a Health Insurance Portability and Accountability Act (HIPAA)–compliant videoconferencing platform in October 2020. The questions presented in the focus groups were coproduced with 4 peer and nonpeer academic scientists and 4 peer support specialists in a community-engaged research approach described as Peer and Academic partnership to help determine important administrative, educative, and supportive guidelines supervisors could use to support the practice of competent digital peer support and support digital peer support specialists [10]. The interview guide contained questions such as the following: “*what essential knowledge does a peer support specialist need to offer digital peer support?*” “*what are the essential abilities peer support specialists need to offer digital peer support?*” “*how do these essential skills vary by lived experience (e.g., mental health, physical health, substance use challenges, Veteran status, aging, racial or ethnic diversity)?*”

The collaborative development of supervision standards is important within social-environmental historical contexts [11]. To reproduce such a group process and promote cross-individual opinions, we used a series of focus groups to develop potential supervision standards and guidelines. The analysis of digital peer support supervision standards was based on four 1-hour focus groups with a total of 59 participants, which took place in October 2020. The focus group discussions followed Morgan's process model [12]. The focus groups were conducted by 2 authors using the interview guide. The interview guide was successfully tested in a pretest. The focus group discussions were recorded digitally, transcribed, and anonymized. Researchers analyzed the data using Rapid and Rigorous Qualitative Data Analysis (RADar), a team-based approach to coding and analyzing qualitative data [13]. This approach was selected because the RADar method produces qualitative results thoroughly and quickly through its ability to organize, reduce, and analyze data in user-friendly software packages such as Excel (Microsoft Corp) [13]. The final set of supervision standards is based on focus-group findings and used member checking to ensure face validity and accuracy. Member checking was used to increase the credibility of participant involvement and data analysis [14]. Researchers presented data transcripts to focus group participants for feedback, and participants were asked to review the transcripts to determine if the researchers' interpretation of the data match their intended meanings [14].

### Analysis

Transcripts were formatted into an all-inclusive Excel sheet that included column headings such as question, participant number, and response. Team members assigned codes to each response. After the all-inclusive Excel sheet was produced, the data table was reduced to include only content relevant to the interview questions. The remaining text and codes were then organized into themes. In accordance with the RADar methodology, themes were determined by the incidence of a code aligned with an overarching theme (see Results). The process of member checking was used to ensure the codes were interpreted correctly and correctly organized into themes. Member checking is a qualitative method used to validate findings, resolve conflicting results, and assess the trustworthiness of qualitative results [15]. The percentage for each theme was found by dividing the frequency in which the theme was present in the focus group quotes by the total number of focus group quotes.

### Ethical Considerations

The Committee for the Protection of Human Subjects at the Dartmouth Health institutional review board approved the project (STUDY02000620). Participants were told in the consent form that they may voluntarily participate in the 1-hour web-based focus group via a HIPAA-compliant videoconferencing platform, and transcripts would be stored via HIPAA-compliant software accessible only to the research team.

## Results

### Participants

A total of 59 peer support specialists participated in the 4 focus groups. Participants' characteristics were reported prior to the interview. The majority of participants were female (35/59, 76%), and the majority of participants had a minimum of high school education (46/59, 100%). Participants were from 11 states and 3 countries.

### Themes Covered

We identified 51 codes and a set of 11 themes related to the development of supervision standards for digital peer support. Themes covered administrative, educational, and supportive functions that participants believed were integral to the supervision of digital peer support specialists. The eleven themes in order of most frequent to least frequent included: (1) education on technology competency; (2) education on privacy, security, and confidentiality in digital devices and platforms; (3) education on peer support competencies and how they relate to digital peer support; (4) administrative guidelines; (5) education on the digital delivery of peer support; (6) education on technology access; (7) supervisor support of work-life balance; (8) emotional support (emerging); (9) administrative documentation (emerging); (10) education on suicide and crisis intervention (emerging); and (11) feedback (emerging).

### Education on Technology Competency

The most prevalent theme was education on technology competency. This core theme constituted 21.8% (43/197) of the themes discussed in the focus groups. Peer support specialists believed it would be important for supervisors and agencies to help digital peer support specialists to develop knowledge on different technological platforms and devices. One peer support specialist mentioned:

A peer support specialist needs to have a comfort with whatever virtual platform they are using to interact with the service user. …I would imagine that, you know, the process of engagement would look very different depending on what kind of technology is being used as the shared platform.

Multiple participants added that digital peer support specialists should not only have basic knowledge of technology but should also be able to teach others how to use technology. One participant said:

They [digital peer support specialists] need some basic knowledge of computers and a virtual format, like accessing applications like zoom, not only to be able to navigate it, but to teach others how to access it and to navigate it on different platforms, like a computer and a mobile phone.

Access to training on digital technology and platforms was encouraged, and some peer support specialists suggested that agencies should offer videos and support in the acquisition of technology knowledge. One peer support specialist mentioned:

We offer different training videos and whatnot for different peer support levels…I think that's something that would be interesting to see rolled out to other organizations as well. Having those sort of videos on how to use the technology exactly how to incorporate your thoughts and empathy into words and how to convey exactly what you're hiding.

### Education on Privacy, Security, and Confidentiality in Digital Devices and Platforms

The second theme, education on privacy, security, and confidentiality in digital devices and platforms, constituted 16.8% (33/197) of the themes discussed in the focus groups. Peer support specialists recommended that agencies and supervisors help to clarify and educate peers on certain topics related to privacy, security, and confidentiality. For example, a few participants suggested education on privacy policies, mandatory reporting, and data collection. One participant questioned:

There's data, so how is data being collected, if it's being shared? Are there third parties? Who's being shared with? Is it shared with treatment teams? Is there data that need is like how does mandated reporting work for certain in certain circumstances with different agencies?...Is there a privacy policy?

Another participant recommended the clarification of definitions of privacy, security, and confidentiality and how they relate to the agency and to the service users in which peer support is being offered. One peer specialist said:

I think definitely, definitely just what the definition of confidentiality means and how it can be defined differently to different individuals…And I think having that mutuality between you what does confidentiality mean to you, what does it mean to me?

Developing knowledge in the evaluation of the security and privacy of digital platforms was not only important to peer support specialists but also having transparency around how privacy, security, and confidentiality guidelines may change depending on the form of peer support. One participant mentioned:

Differentiating between a warm line support call versus a crisis call…let people know this is a warm line. And we can talk about certain things. But as soon as you start to talk about harming yourself or others, now, this is turned into a crisis call. It's no longer a warm line call. And what I've been told by emergency services workers in Virginia, one in particular, is that once there is a crisis, confidentiality, HIPAA no longer applies.

### Education on Peer Support Competencies and How They Relate to Digital Peer Support

The third theme, education on peer support competencies and how they relate to digital peer support, constituted 12.7% (25/197) of the themes discussed in the focus groups. Many peer support specialists emphasized the importance of offering training on general peer support and maintaining the important values and principles of peer support during transitions to digital settings. One participant suggested:

Peer support requires many different skills. What essential knowledge do peer support specialists need to offer digital peer support? They need to know peer support cold. They need to be aware of how that translates virtually.

Other participants discussed the skills they would look for in peer support specialists when hiring them for peer support. They believed that a competent delivery of services would include general peer support competencies. One peer support specialist said:

I would want them to be top-notch peer supporter[s] before we even go into digital peer support. I would want someone that knows peers support, [and] that had the certified peer support specialist or a recovery coach so [he/she/one] knows how to deal with substance use, knows good peer support, and has a solid foundation. We’re not hiring people to do digital stuff; we’re hiring people to do peer support and the digital platform is just one way to deliver the peer support.

While digital peer support requires new knowledge, skills, and supervisor support, it also requires solidification of the basic principles and values of peer support within the digital environment. One participant mentioned:

Peer support is peer support. I’ve been doing this for 23 years, I’ve talked to doctors, nurses, graduate students, etc and they all think that we are the saviors, and we’re going to be able to do the work that they can’t do. I’m just gonna sit down with them, I’m going to shut up, I’m going to listen, and I’m not going to fix them, I’m not going to judge them. So really, peer support, whether it’s digital or face to face, is the same basic principles.

### Administrative Guidelines

The fourth theme was administrative guidelines. The theme of administrative guidelines constituted 10.7% (21/197) of the themes discussed in the focus groups. Peer support specialists recommended the development and transparency of administrative guidelines surrounding topics such as technology security, work-life boundaries, and suicide prevention. One participant said:

I think that's essential knowledge for the peer specialist to know what they are, what boundaries they have, for what roles they may be having, and that they're not all the same.

For example, participants suggested the creation of agency guidelines around communication methods used in suicide prevention on the internet. One participant mentioned, “having a real good fine communication flow chart. So that if an event does happen, that, you know, the peer support will know you know what to say, and also who to reach out to, so that they can get the extra support that they need through this as well” around confidentiality and what information peers need to share with supervisors. One peer support specialist said, “there are certain things that if we discussed and talk about I have to share with my supervisor” and around digital platforms and technologies that are acceptable for digital peer support. For example, one participant observed, “the state mandates what [digital platforms and technology] you can and can’t use when you’re providing services to their peers or to your peers.”

### Education on the Digital Delivery of Peer Support

The fifth theme, education on the digital delivery of peer support, constituted 9.1% (18/197) of the themes discussed in the focus groups. With the transition from in-person peer support to digital peer support, peer support specialists have requested for agencies and supervisors to acknowledge the difference between providing services in-person opposed to digital. Many peer support specialists have appealed for training surrounding digital technologies and the digital delivery of peer support. For example, one participant mentioned:

I would want them to attend the digital peer support training. I think some training in digital support, the separate platforms and just talking about software and hardware and how they work together.

This includes training and skill development to engage service users on the internet and display empathy in a digital setting. One peer support specialist suggested:

Some sort of technical training on empathetic listening or some other ways of being able to convey emotion without necessarily being able to be connect. A personal conversation training maybe.

Participants also recommend that supervisors provide peer support specialists with the opportunity to decide whether digital peer support versus in-person peer support would be the best option for them, based on knowledge of both roles. One participant said:

Make sure any new hiring you’re having do digital support want to be a part of that. Not just ‘okay, we’ve hired you as a peer and you’re going to do this also’ because for some people it would be uncomfortable for them to be providing those services digitally.

### Education on Technology Access

The sixth theme, education on technology access, constituted 8.6% (17/197) of the themes discussed in the focus groups. Access to technology and internet service is an important aspect of digital peer support. However, there are many populations that have difficulties accessing the devices and infrastructure they need to effectively use digital peer support. In order to expand the reach of digital peer support, peer support specialists believe peers need to be aware of the resources and tools available for the support of underserved populations. For example, one participant said:

it takes a really, really strong commitment and awareness to really open the doors of the service system much, much wider. Essentially, now that you know, there's so much construction to the pandemic right.

Supervisors could potentially help digital peer support specialists to gain knowledge on resources and tools necessary to meet the needs of individuals, such as those experiencing homelessness or those living in rural settings. On participant mentioned:

There are a lot of individuals who may be on it, whether they're experiencing homelessness, or don't have consistent access to, for example, like charging or maybe have like, very, very basic phone, like flip phones. And so I would think to having kind of resourcefulness maybe being…having tools to be able to, I think, be proactive and kind of have insight and awareness to meet the needs of various populations in terms of location and in access.

### Supervisor Support of Work-Life Balance

The seventh theme, supervisor support of work-life balance, constituted 8.6% (17/197) of the themes discussed in the focus groups. Supervisor support is essential to the maintenance of a healthy work-life balance. Many peer support specialists believe supervisors can help to set time limits and boundaries in service user’s access to telehealth. One participant recommended:

Incorporate, you know, some boundaries around your personal time, and to make it a main priority. And again, I think with the accessibility of the, of telehealth, it just is, you know, it just makes it just that much more important. … the supervisor is going to have to help out with this.

Supervisors should be mindful of digital fatigue and help peer support specialists to schedule adequate breaks and time-limits. For example, one peer support specialist recommended:

…setting reasonable limits as to the amount of time that is expected of people. So giving people adequate breaks and stuff, so they don't have to do too much. …just being mindful of when you are starting to get that fatigue.

Open communication and collaboration between supervisors and peer support specialists can help to address and resolve issues with work-life balance. One participant said:

…be upfront and open with those that you're supporting of your availability, because it's also important for you to protect yourself and your boundaries and maintain your health and self-care and all those things.

### Emotional Support

The eighth emerging theme, emotional support, constituted 4.6% (9/197) of the themes discussed in the focus groups. Peer support specialists believe the additional challenges that come with digital peer support require additional support. Many participants believed reaching out for help from a supervisor or other peer can not only help with self-care but can help the peer support specialists to grow in their role as a digital peer support specialist. One participant said:

I think an essential ability is to that when you are having those tough moments to make sure that people reach out for help, so that they don't feel like they're struggling on their own, and that they do get feedback on some of those maybe tougher cases… don't be afraid to reach out for help, because it, it helps you grow in your role. But it also helps with the self-care.

Many participants agreed that the transition to digital peer support was often stressful and overwhelming. For example, 1 peer support specialist said:

Whether it's a digital or, you know, traditional, but I think more so digital, because a lot of us peer supports are new at this to, like, you know, it was hard enough for us to document now, and now we're trying to like, enter, you know, and do all this stuff on a phone or a computer, that is just a more stressful and I think it just makes me think of it, there's peers that have kind of, you know, resigned because of that, because it's like, Ah, you know.

As a result, many believed emotional supervisor support could help peer support specialists to address feelings such as stress and fatigue and other experiences such as retraumatization. One participant mentioned:

With peer support, you know, we talk about the trauma informed, because it's a part of our lived experience to share those pieces where it builds connection, we might get re traumatized all over again, and not even realize it until we're trying to sleep that night that something doesn't feel right. You know, and so to be able to talk about it to reach out to your supervisor or another coach or peer, I think is really important and that's foundational.

### Administrative Documentation

The ninth theme, administrative documentation, was an emerging theme and constituted 3% (6/197) of the themes discussed in the focus groups. Participants agreed that documentation of peer support would differentiate between in-person and web-based support. One participant mentioned, “I think documentation is going to be a little different for what you have to do on telehealth versus what you’re doing in person.” Knowledge of the changes in documentation requirements and regulations is important to the supervisor role. For example, 1 participant suggested knowledge in “certain regulations…or documentation” and “knowing kind of what with your state are certain requirements or different kind of policies at the state level.” Participants suggested that digital peer support and the use of digital devices and platforms may offer challenges to the process of documentation for supervisors and others. One peer support specialist said, “It was hard enough for us to document and now we’re trying…to do all this stuff on a phone or computer.”

### Education on Suicide and Crisis Intervention

The tenth emerging theme, education on suicide and crisis intervention, constituted 2.5% (5/197) of the themes discussed in the focus groups. Many participants emphasized the difference between suicide and crisis intervention in-person opposed to digitally. For example, 1 participant said, “we're all very skilled in in-person crisis response and that completely changes when you're digital.” As a result, peer support specialists believe supervisors should offer trainings on digital suicide and crisis intervention. One participant recommended:

A training [or] even just a conversation about how to respond to someone that is in crisis, virtually. When you can't be there to control what's in their environment or their actions, how can you respond to keep them safe while you get them in person assistance? I think that's an important discussion that needs to be had and maybe training that needs to be developed.

Digital peer support specialists should know when to contact supervisors in an emergency. For example, 1 peer support specialist suggested the importance of “making sure that somebody is sufficient in those competencies such as suicide prevention, how to contact supervisors in a[n] emergency, how to diffuse the situation, [and] talk somebody down” and have access to skill development and trainings in crisis intervention. One participant said:

One of the things that you would need to know is when to activate and do an active rescue versus just an imminent risk. If you’re going to take on that role, there’d have to be some additional training of knowing when it is [a] crisis, [or] that they’re just reaching out for help and support.

### Feedback

The last emerging theme, feedback, constituted 1.5% (3/197) of the themes discussed in the focus groups. Many peer support specialists believe receiving feedback from supervisors is important to building competency in digital peer support specialists. One participant suggested supervisor feedback is helpful in “practicing how to do the things that you talk about doing. And making sure that somebody is sufficient in those competencies such as suicide prevention, how to contact supervisors in case there is a need for emergency, how to defuse the situation, how to talk somebody down although that's the same thing with diffusing situation or what I'm saying and yeah that's about it.”

Feedback also helps to uncover skills or knowledge supervisors themselves may need to improve upon. For example, 1 peer support specialist suggested that feedback “helps you as a supervisor to know what skills you need to work on both in supervision and you know for trainings.” Peer support specialists recommended feedback methods such as having a supervisor sit in on a meeting or call, or practicing role play in which the supervisor pretends to be in a crisis and then discusses the ways in which the specialist could have improved their digital support. One participant recommended having “the supervisor sit in on one or two or three or four of the peer support groups or calls” and another recommended having “a fake call or fake message conversation where for an hour the supervisor pretends to be in crisis and reaches out and we have to provide ample support to them and then they critique us on everything that we said after the hours up and they tell us if we are allowed to go on and do peer support provider.”

## Discussion

### Principal Findings

The following themes emerged from the four focus groups (N=59): (1) education on technology competency; (2) education on privacy, security, and confidentiality in digital devices and platforms; (3) education on peer support competencies and how they relate to digital peer support; (4) administrative guidelines; (5) education on the digital delivery of peer support; (6) education on technology access; (7) supervisor support of work-life balance; (8) emotional support (emerging); (9) administrative documentation (emerging); (10) education on suicide and crisis intervention (emerging); and (11) feedback (emerging). Established supervision standards may help to promote the competent delivery of digital peer support and help to encourage skill development, knowledge, support, and guidelines for the digital peer support role. These supervision recommendations may act to enhance the established supervision standards endorsed by SAMHSA.

The purpose of this study was to inform the development of supervision standards for digital peer support and introduce guidelines that supervisors can use to support, guide, and develop competencies in digital peer support specialists. Currently, supervision standards from SAMHSA for in-person peer support include the categories of administrative, educative, and supportive functions. However, the spread of digital peer support during the COVID-19 pandemic requires the expansion of supervision standards to include subthemes such as education on technology and privacy, support of work-life balance, and emotional support. Without digital supervision standards, there are potential risks of a breach in ethics and confidentiality, workforce stress, loss of productivity, loss of boundaries, and ineffectively serving users who participate in digital peer support services.

Digital peer support is quickly expanding across the globe. However, the transition to digital peer support brings new challenges and the necessary acquisition of new guidelines and skills. While SAMHSA has developed supervision guidelines for in-person peer support, digital peer support requires the expansion of supervision standards and the significance of administrative, educative, and supportive supervision. Digital peer support specialists require specific knowledge and skills to communicate with service users and deliver peer support effectively. Therefore, supervisors also require new knowledge and skills to effectively develop, support, and manage the digital peer support role.

### Limitations

This study is not without limitations. First, there are potentially other supervision standards and guidelines that have not been identified. Second, the sample lacked diversity based on racial and ethnic background. Future studies should consider the inclusion of disadvantaged populations such as Hispanic, and LGBTQIA populations and demographics such as homeless individuals. Third, as technology changes and digital peer support expands, supervision standards will need to be updated. Fourth, the findings cannot be generalized to all digital peer support specialists due to the small sample size. However, we reached saturation when additional data did not provide more information across international boundaries, and the themes identified could be used to promote consistency in the practice of digital peer support. Fifth, the data could not be stratified by volunteer-run services versus paid professional services or the role of the participant [16]. Sixth, recruitment occurred via a peer support specialist listserv—not solely service users of the mental health system. Including the voices of service users can enhance these competencies. Lastly, focus groups with specified digital peer support supervisors could help to expand the findings. Future research looking at the integration of digital peer support competencies within digital peer support supervision is needed. Future research should work to verify and build off of the digital peer support supervision standards and guidelines defined in this manuscript.

### Comparison With Prior Work

Prior work has shown that digital support can be just as effective as in-person support in patient-clinician engagement [17]. There are, however, a few concerns about using the digital environment to facilitate health care. Digital health care may lead to social isolation without proper design in mHealth interventions [18]. Social isolation could be caused by the optional nature of interfacing with others when using technology and inability to connect using visual insights and nonverbal cues [19]. Prior work has expressed the importance of training health care workers on demonstrating digital empathy to address differences from in-person interaction [19]. Additional security must also be implemented in mHealth interventions including HIPAA compliance in videoconferencing software and digital patient records, to ensure privacy and confidentiality [20]. Therefore, digital peer support may increase the capacity for engagement with individuals while providing quality relationships and satisfactory care if supervision standards are improved to accommodate digital security and address the potential for concerns such as social isolation and empathy [5]. In addition to improving quality of care, proper supervision may facilitate peer recovery specialist practice. In recent peer recovery specialist literature, it was expressed in focus groups that consistent supervision that emphasizes self-care and principles learned in training may lead to greater worker retention and job satisfaction [21].

### Conclusions

Introducing supervision standards for digital peer support is a first step in helping to guide the delivery of digital peer support and the development of digital peer support specialists. As defined by SAMHSA, supervision is important to competency building and skill development in peer support. Supervision has the potential to improve performance, empower workers, and promote knowledge of the peer role. The shift to digital peer support has expanded the reach of in-person support and has shifted how peer support is both delivered and managed. The identification of supervision guidelines for digital peer support has the potential to facilitate the transition from in-person to digital peer support and promote best practices in both digital peer support delivery and the supervision of digital peer support specialists.

## Abbreviations

HIPAA: Health Insurance Portability and Accountability Act
RADar: Rapid and Rigorous Qualitative Data Analysis
SAMHSA: Substance Abuse and Mental Health Services Administration
